# Genetic mapping of principal components of canine pelvic morphology

**DOI:** 10.1186/s40575-017-0043-7

**Published:** 2017-03-24

**Authors:** Mark J. Fealey, Joy Li, Rebel J. E. Todhunter, Ursula Krotscheck, Kei Hayashi, Marina J. McConkey, Adam R. Boyko, Jessica J. Hayward, Rory J. Todhunter

**Affiliations:** 1 Department of Clinical Sciences, College of Veterinary Medicine, Cornell University, Ithaca, NY 14853 USA; 2 Department of Biomedical Sciences, College of Veterinary Medicine, Cornell University, Ithaca, NY 14853 USA; 3 Cornell Veterinary Biobank, College of Veterinary Medicine, Cornell University, Ithaca, NY 14853 USA

**Keywords:** Dog, GWAS, Principal component analysis, IGF-1, Pelvic sexual dimorphism, Norberg angle, Hip dysplasia

## Abstract

**Background:**

Concentrated breeding effort to produce various body structures and behaviors of dogs to suit human demand has inadvertently produced unwanted traits and diseases that accompany the morphological and behavioral phenotypes. We explored the relationship between pelvic conformation and canine hip dysplasia (HD) because purebred dogs which are predisposed, or not, to HD share common morphologic features, respectively. Thirteen unique bilateral anatomical features of the pelvis were measured on 392 dogs of 51 breeds and 95 mixed breed dogs. Principal components (PCs) were derived to describe pelvic morphology. Dogs were genotyped at ~183,000 single nucleotide polymorphisms and their hip conformation was measured by the Norberg angle and angle of inclination between the femoral neck and diaphysis.

**Results:**

No associations reached genome wide significance for the Norberg angle when averaged over both hips. PC1 was negatively correlated with the Norberg angle (*r* = -0.31; *P* < 0.05) but not the angle of inclination (*r* = -0.08; *P* > 0.05). PC1, 2, 4, and 5 differed significantly between male and female dogs confirming pelvic sexual dimorphism. With sex as a covariate, the eigenvector contribution to PC1 reflected the overall size of the pelvis and was significantly associated with the *IGF-1* locus, a known contributor to canine body size. PC3, which represented a tradeoff between ilial length and ischial length in which a longer ischium is associated with a shorter ilium, was significantly associated with a marker on canine chromosome 16:5181388 bp. The closest candidate gene is *TPK1*, a thiamine-dependent enzyme and part of the *PKA* complex. Associations with the remaining PCs did not reach genome wide significance.

**Conclusion:**

*IGF-1* was associated with the overall size of the pelvis and sex is related to pelvic size. Ilial/ischial proportion is genetically controlled and the closest candidate gene is thiamine-dependent and affects birth weight and development of the nervous system. Dogs with larger pelves tend to have smaller NAs consistent with increased tendency toward HD in large breed dogs. Based on the current study, pelvic shape alone was not strongly associated with canine hip dysplasia.

**Electronic supplementary material:**

The online version of this article (doi:10.1186/s40575-017-0043-7) contains supplementary material, which is available to authorized users.

## Plain English Summary

Concentrated breeding effort to produce various body structures and behaviors to suit human demand has inadvertently produced unwanted traits and diseases that accompany the external appearance and behavior of dogs. Purebred dogs, which are predisposed, or not, to HD share common features of their shape and size, respectively. Thirteen unique anatomical features of the pelvis were measured on radiographs of 392 dogs of 51 breeds and 95 mixed breed dogs. Combinations of these measurements together described the shape and size of the pelvis. Male dogs had significantly larger pelves than female dogs. Genetic markers pointed to insulin-like growth factor-1 as a major driver of pelvic size. A second genetic marker was associated with ilial length and ischial width on canine chromosome 16. Conclusion: Based on the current study, pelvic shape alone was not strongly associated with canine hip dysplasia.

## Background

The domestic dog is arguably the most morphologically diverse mammal [1]. The vast differences in morphology within the species have suggested that genetic variation can rapidly change anatomical features, some of which are related to deleterious traits. Breeds as diverse as Chihuahuas, Great Danes, Salukis, and Bulldogs are all descended from the gray wolf, and are the product of selection that began when the dog derived from the wolf about 15,000 years ago but exact timelines remain elusive [2]. The formation of modern dog breeds began about 200 years ago [3]. The speed and coherence with which these functional adaptations have occurred suggests that selection may be acting on genetic loci that control multiple morphological structures.

The selection of certain morphologic features is likely correlated with the selection of genes that predispose dogs to orthopedic diseases. Breeds such as the American Bulldog and Saint Bernard, which are large and stocky, have an increased propensity to develop hip dysplasia (HD) than breeds such as the Greyhound, Saluki or Borzoi [4]. Hip dysplasia is the abnormal development of the coxofemoral joint(s). Joint laxity is generally considered to be one of the earliest pathologic findings in HD and is a major precursor for the osteoarthritic changes that are typically associated with HD [5, 6]. The precise genetic factors that initiate HD are unknown and the rate and extent of its development are variable. Since the first report of HD in the dog in 1935, the disorder has become one of the most commonly diagnosed canine orthopedic diseases [7].

The polygenic mode of inheritance of HD has made reduction in its prevalence slow [8]. The inability to identify the specific genes responsible for the predisposition to HD has, until recently, left only phenotypic evaluation by radiography for the screening of individuals. The expression of polygenic traits is modified by environmental influences thus reducing the proportion of phenotypic variance that has a genetic basis [8]. Heritability of HD ranges from 0.2 to 0.6 [9–13]. Because morphology and orthopedic disease are intertwined, probing the genetic basis of pelvic morphology may shed light on the genetic basis of the HD here measured by the Norberg angle.

Chase et al., [14] described the relationships of what they referred to as “tradeoffs” in pelvic morphology of the Pit Bull Terrier type dog and the Greyhound. When the ratio of the length of the long bones to the width of the cranium and the ratio of the muscle mass of the hind limbs to the diameter of the femur increases, the susceptibility to HD decreases. Morphotypes like the Pit Bull Terrier and American Bulldog are predisposed to HD while Greyhounds, Borzois and Salukis are less susceptible to HD (http\offa.org) [9, 15]. Because dogs with HD have disease in other joints [16–19] and the acetabulum is part of the pelvis, it behooves the question: are there morphological features of pelvic shape that contribute to HD? Chase et al., [14] point out that quantitative trait loci (QTLs) related to pelvic shape may be relevant to disease. They found that a particular haplotype of the QTL associated with the simple sequence repeat marker *FH2388* was associated with osteoarthritis (OA) in the coxofemoral joint that resulted from HD in Portuguese Water Dogs. This marker, they also discovered, was associated with pelvic shape. They proposed that the acetabular OA appeared to result directly from the action of the QTL haplotype rather than indirectly from changes in pelvic shape produced by the QTL [20].

To assess the relationship of the femoral head and proximal femur with the acetabulum and pelvis, we measured the Norberg angle and the angle of inclination of the femoral neck to the femoral diaphysis in dogs. The Norberg angle [21] measures the coverage of the femoral head by the acetabulum with the femora in an extended position and the dog lying in dorsal recumbency. The angle of inclination has been used to assess normal and dysplastic conformation in dogs but its relationship to HD has been equivocal [22–25].

One method to explore the relationship between many measurements on the same organ is through principal component analysis (PCA), which classifies phenotypic variation into independent systems of correlated traits [26]. Individual dogs each have a value for every principal component (PC). Principal components of pelvic shape are heritable [14]. Thus, PCs are phenotypes that can be subjected to genetic analysis, and QTLs can be identified that inform these phenotypes. As Chase et al., [14] elegantly explained, the genetics of PCs can be used to dissect genetic networks that regulate complex biological systems like pelvic shape [14] and in so doing, we may discover variants that contribute to HD or protect against it.

The aims of this study were to use PCA to reduce morphologic features of the canine pelvis into a set of independent variables and then to map these PCs in a genome wide association study. Our hypothesis was that QTLs would be associated with the PCs of these pelvic measurements. Genes in these QTLs may contribute to HD because the coxofemoral joint is an integral part of the pelvic architecture so that pelvic conformation and HD are inextricably linked.

## Methods

### Dogs

Pelvic dimensions were measured from digital or hard copy radiographs from the following sources: the Cornell University Hospital for Animals, the Baker Institute for Animal Health at Cornell University, and the Guiding Eyes for the Blind in Yorktown Heights, NY. A total of 530 dogs were measured but all measurements were available on 392 dogs, which formed the cohort for this study. The majority of which were German Shepherd (6% [*n* = 25]), Golden Retriever (9% [n = 36]), Labrador Retriever (35% [*n* = 137]), and Greyhound/ Labrador Retriever cross breed dogs (24% [*n* = 95]) (Table 1).

**Table 1:** Breed summary of Norberg Angle (NA) in degrees expressed as the mean (standard deviation)

Breed	No. dogs	Left NA	Right NA	Average NA
German shepherd dog	25	91.5 (11.7)	92.3 (11.2)	91.9 (10.7)
Golden retriever	36	92.1 (10.5)	92.9 (12.6)	92.3 (11.3)
Labrador retriever	137	103.8 (9.2)	104.4 (9.2)	104.1 (8.8)
Newfoundland	15	91.1 (11.5)	96.9 (9.2)	94.0 (9.9)
Labrador/Greyhound cross	95	106.8 (5.8)	107.7 (4.9)	107.2 (4.7)
Other	82	96.6 (12.2)	98.9 (12.3)	97.8 (11.9)
Total	397			

### Radiographic measurements

**Fig. 1:**
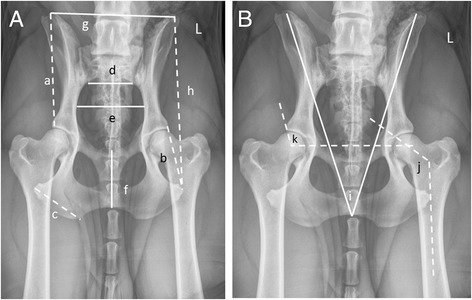
**a** Ventrodorsal radiograph of a pelvis with measurement labels for ilial length (*a*), ischial length (*b*), ischial tuberosity length (*c*), width of sacrum (*d*), pelvic inlet diameter (*e*), pubis to ischium (*f*), cranial ilial span (*g*), and os coxa length (*h*). **b** This image shows the internal pelvic angle (*i*), angle of inclination (*j*), and Norberg angle (*k*). For abaxial measurements, both the *left* and *right* sides were measured and bilateral measurements are indicated by *dashed lines*

Thirteen pelvic dimensions (pubis to ischium, left ischial length, right ischial length, span of cranial ilium, width of sacrum, left ilial length, right ilial length, left ischial tuberosity length, right ischial tuberosity length, left os coxa length, right os coxa length, pelvic inlet diameter, internal pelvic angle) were measured in millimeters on a pelvic radiograph, with the dog in one of four different positions: ventrodorsal extended hip (Fig. 1), ventrodorsal frog-leg, ventrodorsal PennHip™, or the dorsolateral subluxation position. Forty randomly selected radiographs were remeasured to assess repeatability of the measurements. The measurements were summarized descriptively using the stat.desc function in the pastecs package in R [27]. The Norberg Angle (NA) was measured for each hip of these dogs, and then averaged. All NA scores below 75° were set to 75° to reduce outlier effects. Hip dysplasia was also trichotomized, based on the average Norberg angle, as normal (Norberg angle > 105°), indeterminate (95°-105°), and dysplastic (<95°) conformation [28]. The angle of inclination was measured on each femur of each dog from the ventrodorsal extended hip radiograph, and then averaged for each dog.

### Principal component analysis

The prcomp function in R [27] was used to calculate PCs from the 13 measured hip phenotypes for 392 dogs of 51 different breeds and 95 mixed-breed dogs. The 392 dogs had radiographic measurements for all 13 phenotypic traits. The correlation matrix was used in the PCA to account for the different dog sizes, scales, and magnifications of the radiographic measurements.

Body weights (expressed as body weight^0.303^ based on a Box-Cox transformation to normalize the distribution of weights across breeds) were available for 188 of the 392 dogs. We regressed body weight against each PC to determine significant relationships in this group of dogs. Correlation analysis was also used to determine if the average Norberg angle or the average angle of inclination was related to any of the PCs.

### Genome-wide association study

The dogs used in the pelvic PCA were genotyped as part of a complex trait mapping study [29]. Briefly, genotyping was done using the Illumina 170k CanineHD array, with the addition of 12,143 custom markers (see PLINK genotype files used in that paper by Hayward et al. [29] that are deposited in Dryad) producing an array composed of ~183,000 markers. The genotyping methods are exactly as described [29]. Using PLINK v1.07 [30], 180,117 single nucleotide polymorphisms (SNPs) remained after filtering (removal of SNPs with a genotyping rate <95%, that deviated from Hardy-Weinberg equilibrium, or that were discordant between duplicate samples), with an overall call rate of >99.8%. Phasing was done for all autosomal and X chromosome markers, and additional custom plates or CanineHD datasets were pre-phased using SHAPEIT [31], and then phased with IMPUTE2 [32].

For the genome wide association study (GWAS), the PCs, the Norberg angle averaged over each dog, and the angle of inclination averaged over each dog were analyzed in a linear mixed model framework using the program GEMMA v.0.94 [33]. Only SNPs with minor allele frequency (MAF) > 0.05 were included in the analysis and a significance threshold of *P* < 3.5×10^−7^ (the Bonferroni-adjusted genome wide *P*-value < 0.05) was used. Because pelvic sexual dimorphism has been described in dogs previously [34], we used a *t*-test to determine if there was a difference between the sexes for each PC, and then included sex as a covariate in the GWAS for those PCs that were significantly different (*P* < 0.05) according to sex.

## Results

### Dogs

The 13 radiographic measurements are summarized in Table 2. The Norberg angle and angle of inclination were analyzed separately for their relevance to hip conformation and HD and were not included in the PCA. The Norberg angle and the internal pelvic angle were significantly correlated (*r* = -0.17, *P* < 0.001), but the angle of inclination was not significantly correlated with either the Norberg angle or the internal pelvic angle (*r* = 0.042, *P* = 0.21 and *r* = 0.048, *P* = 0.18, respectively). The correlation coefficient between the five bilateral measurements ranged from 0.6 to 0.96. The angle of inclination correlation between left and right was 0.6. This is not surprising because each hip of each dog varies by natural asymmetry and HD. For 20 randomly selected repeat digital radiographic measurements, the correlation coefficients ranged from 0.69 to 0.97. For 20 randomly selected repeat hard copy radiographic measurements, the correlation coefficients ranged from 0.42 to 0.98. Two dogs had the original right and left os coxa lengths used for the respective ilial lengths reducing the correlation coefficient to 0.42. Thus two dogs had incorrect individual eigenvalues for PC3. The pelvic measurements of all 530 dogs are provided in Additional file 1: Table S1 and the subset of 392 dogs in Additional file 2: Table S2.

**Table 2:** Descriptive statistics summary of the 13 radiographic measurements (mm) shown in Fig. 1

	pubis to ischium (f)	left ischial length (b')	right ischial length (b)	span of cranial ilium (g)	width of sacrum (d)	left ilial length (a')	right ilial length (a)
Min	23.1	27.5	28.7	59.0	24.0	50.4	51.9
Max	67.4	106.9	105.8	152.9	65.0	152.0	152.0
Mean	46.1	57.1	57.7	110.1	45.6	101.8	101.0
Median	46.0	56.1	56.3	109.6	45.0	101.0	100.6
Std dev	7.1	8.7	8.9	14.8	6.4	12.3	12.4
	left ischial tuberosity length (c')	right ischial tuberosity length (c)	left os coxa length (h)	right os coxa length (h')	pelvic inlet diameter (e)	internal pelvic angle (i)	
Min	22.1	24.2	84.8	61.0	30.7	29.0	
Max	66.9	74.4	232.7	226.7	85.7	43.0	
Mean	47.1	46.8	155.1	154.5	60.4	36.2	
Median	46.4	45.4	152.8	152.1	59.2	36.0	
Std dev	5.7	5.7	19.9	20.3	8.0	2.4	

The corresponding identifiers for each measurement in Fig. 1 are included next to each measurement. The corresponding bilateral measurement is indicated by the letter prime. All bilateral measurements are indicated as dashed lines in Fig. 1

### Principal component analysis

The first eigenvector explained 72% of the overall variance and the first four vectors explained 90% of the overall variance (Table 3). The first PC was composed about equally of all the pelvic measurements except the internal pelvic angle (Fig. 1), which was the major contributor to PC2 (Table 4). Body weight was strongly correlated with PC1 (*r* = 0.79, *P* < 0.0001) and weakly correlated with PC2 (*r* = 0.14, *P* = 0.02), PC7 (*r* = 0.21, *P* = 0.002) and PC8 (*r* = 0.18, *P* = 0.007). The breeds roughly clustered by body weight (PC1) (Additional file 3: Figure S1A and B). We further showed that the major breeds can be distinguished based on their PC1 values but not based on their PC3 values (Additional file 4: Figure S2). Therefore, body size is a major contributor to PC1 but not to PC3.

**Table 3:** Eigen values, variances, and cumulative variances for the principal components (PCs) of 13 pelvic measurements summarized in Table 2

Principal component	Eigenvalue	Variance	Cumulative variance
PC1	9.39	72.19	72.19
PC2	1.15	8.86	81.05
PC3	0.65	4.97	86.02
PC4	0.52	4.00	90.02
PC5	0.34	2.65	92.67
PC6	0.29	2.21	94.88
PC7	0.19	1.47	96.35
PC8	0.18	1.38	97.73
PC9	0.10	0.78	98.50
PC10	0.09	0.72	99.22
PC11	0.05	0.37	99.59
PC12	0.04	0.34	99.93
PC13	0.01	0.07	100

**Table 4:** Composition of the 13 principal components (PCs) with each individual pelvic measurement weighting

Measurement location	PC1	PC2	PC3	PC4	PC5	PC6	PC7	PC8	PC9	PC10	PC11	PC12	PC13
Pubis to Ischium	0.28	0.02	0.32	0.04	−0.04	0.66	0.44	−0.36	0.01	−0.22	−0.04	0.055	0.01
L.Ischium	0.21	0.14	0.44	0.18	0.11	−0.33	0.10	0.21	−0.13	−0.04	0.33	0.58	0.18
R.Ischium	0.29	0.12	0.44	0.21	0.12	−0.29	0.03	0.15	−0.09	−0.09	−0.50	−0.50	−0.16
Span of Cranial Ilium	0.30	−0.22	−0.14	0.11	0.13	0.18	0.16	0.15	−0.15	0.81	−0.20	0.10	0.04
Width of Sacrum	0.27	−0.17	−0.10	0.01	−0.88	−0.24	0.23	0.05	0.07	−0.01	−0.01	−0.03	−0.00
L.Ilium	0.29	0.19	−0.47	0.10	0.16	−0.07	0.22	−0.01	−0.38	−0.19	0.24	−0.35	0.47
R.Ilium	0.28	0.21	−0.51	0.09	0.18	−0.11	0.15	−0.01	0.13	−0.25	−0.30	0.38	−0.47
L.Ischial Tuberosity	0.28	0.045	−0.01	−0.45	−0.03	0.37	−0.20	0.71	0.05	−0.19	0.02	−0.03	0.01
R.Ischial Tuberosity	0.25	0.03	0.09	−0.80	0.13	−0.31	0.06	−0.39	−0.00	0.14	−0.05	0.01	0.03
L.Oscoxa	0.32	0.06	0.02	0.11	0.04	0.02	−0.17	−0.10	0.13	0.18	0.64	−0.31	−0.53
R.Oscoxa	0.31	0.09	−0.05	0.18	0.06	0.00	−0.31	−0.15	0.73	0.05	−0.09	−0.02	0.45
Pelvic Inlet Diameter	0.29	−0.22	−0.02	0.10	−0.13	0.11	−0.68	−0.29	−0.46	−0.14	−0.14	0.16	−0.00
Internal Pelvic Angle	0.10	−0.86	−0.02	0.02	0.30	−0.14	0.15	0.06	0.14	−0.29	0.08	−0.04	−0.01

Each measurement is shown on radiographs in Fig. 1

**Fig. 2:**
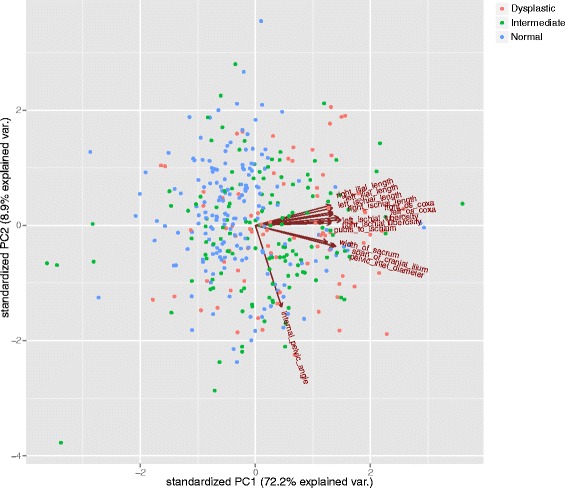
Plot of PC1 and PC2 values for dysplastic (*red*) and normal (*blue*) dogs and for dogs with the intermediate phenotype (*green*)

Consistent with pelvic morphology displaying sexual dimorphism [34], male PC1 was significantly higher than female PC1 (t = 4.35, *P* < 0.0001). When each PC was plotted against another as a function of HD classified as dysplastic, normal, and indeterminate by the Norberg angle groupings, there was no clustering or differentiation between the three classes according to the PC axes (Fig. 2). Male dogs also had significantly different PC2 (t = 2.77, *P* = 0.006), PC4 (t = 2.69, *P* = 0.007) and PC5 (t = 2.12, *P* = 0.034) values compared to female dogs. Thus, sex was included as a covariate for the GWAS modeling of PC1, 2, 4, and 5. PC1 (*r* = -0.31, *P* < 0.0001) and PC7 (*r* = 0.30, *P* < 0.0001) and to a lesser extent PC2 (*R* = 0.09, *P* = 0.043), had modest, but significant, correlations with the NA. Only PC7 was significantly correlated with average angle of inclination (*r* = 0.23, *P* < 0.0001).

The contribution of each measurement to PC3 showed that it represented a tradeoff between ilial length and the length of the ischiatic tuberosity; dogs with shorter ilial lengths have longer/wider ischiatic tuberosities and vice versa. The length of the left and right ischial tuberosities was the major contributor to PC4, sacral width to PC5, length of the pubis to PC6, and pelvic inlet diameter to PC7. Principal components 8 and 9 were reflective of right and left pelvic asymmetry. Principal component 10 was composed predominantly of the span of the cranial ilium.

### Genome wide association study

**Fig. 3:**
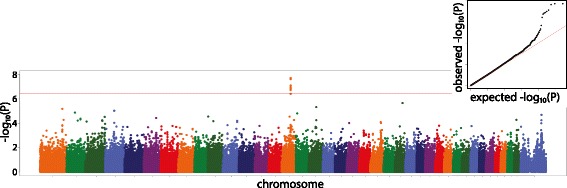
Manhattan plot of genome wide association study of PC1 modeled with sex as a covariate. Marker position across the chromosome is plotted on the X axis against -log_10_(*P*) on the Y axis. The Bonferroni adjusted genome wide threshold is the *solid red line* drawn across the plot. QQ plot of expected versus observed –log_10_(*P*) is also shown as insert

No associations were found for the Norberg angle averaged over both hip joints or the angle of inclination averaged over both hip joints at a genome wide level of significance. Associations with two PCs reached genome-wide significance (Table 5). With sex as a covariate, GWAS for PC1 had the strongest association at 15:41,229,597 bp, which is the locus for *IGF-1* (*P* = 1.9×10^−8^) (Fig. 3). *IGF-1* is a major growth regulator. Supported by the significant relationship between sex and body weight, PC1 reflects overall size of the pelvis.

**Table 5:** Results from genome wide association study performed on the first eight principal components

Principal component (PC)	Chr	Marker position (bp)	MAF	Beta	*P* value
PC1^a^	15	41229597	0.17	−1.34	1.93 × 10^−8b^
PC2^a^	26	15666332	0.05	−0.80	5.75 × 10^−7^
PC3	16	5181388	0.22	−0.35	1.91 × 10^−7b^
PC4^a^	5	36523896	0.04	−0.62	2.47 × 10^−6^
PC5^a^	1	91463706	0.27	0.22	6.67 × 10^−7^
PC6	34	5001347	0.09	−0.33	3.51 × 10^−7^
PC7	26	8569325	0.28	−0.15	1.79 × 10^−5^
PC8	31	29430539	0.07	−0.32	8.88 × 10^−7^

^a^with sex included as a covariate

^b^significant after Bonferroni correction (alpha = 0.05; *P* < 3.5×10^−7^)

Chr is the chromosome number; bp is base pairs; MAF is minor allele frequency; Beta is the test statistic of the Wald test for the strength of the association

**Fig. 4:**
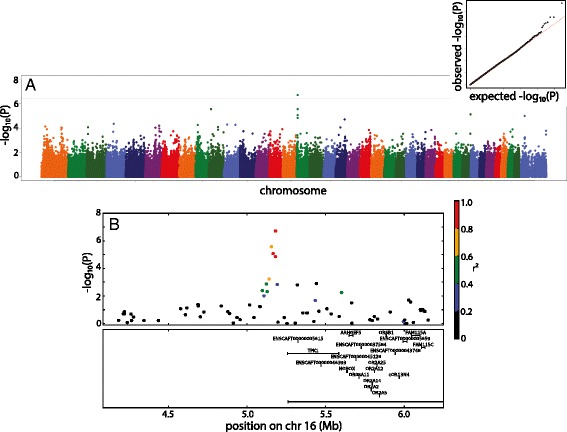
**a** Manhattan plot of genome wide association study of PC3. Marker position across the chromosome is plotted on the X axis against -log_10_(*P*) on the Y axis. The Bonferroni adjusted genome wide threshold is the *solid red line* drawn across the plot. QQ plot of expected versus observed –log_10_(*P*) is also shown as insert. **b** Manhattan plot of 2mb region surrounding the associated SNP. *Colors of dots* indicate the amount of linkage disequilibrium (as measured by r^2^) with the associated SNP

The strongest association with PC2 was at 26:15,666,332 (*P* = 5.8×10^−7^), which did not reach genome-wide significance. GWAS of PC3 yielded a significant association at 16:5,181,388 (*P* = 1.9×10^−7^) (Fig. 4a). This association is located 76 kb upstream of the candidate gene thiamine pyrophosphokinase 1 (*TPK1*) (Fig. 4b). Principal Components 4, 5, 6, 7 and 8 were not associated significantly with any loci, although the association with PC7 was just on the Bonferroni adjusted level of genome wide significance.

Even though the QQ plots showed that the likelihood of false positive associations was well controlled as shown in Fig. 3, we also undertook the GWAS after removing the most related dogs. We removed all Labrador Retriever, Greyhound and Greyhound/Labrador Retriever cross (mix) breed dogs that were related (pi-hat > 0.5), leaving only 25 of these 167 dogs. We then performed the PCA with these related dogs removed and repeated the GWAS (Additional file 5: Table S3). As shown, the PC3 association fell above the threshold for genome-wide significance, but this is not surprising given that this association is driven by Labrador Retrievers and the Greyhound/Labrador Retriever cross-breed (mix) dogs (Table 6). In contrast, the association with the PC1 variant is driven by the breeds other than the major breeds (Table 6).

**Table 6:** This table shows details of the breeds contributing to the strength of the association for PC1 on CFA15 (A) and PC3 on CFA16 (B), in the five main breeds and remaining dogs (other)

(A) chr15:41229597						
BREED	N	Beta	SE	T statistic	*P* value	Freq_G_allele
Golden Retriever	36	1.223	0.833	1.468	0.151	0.056
Labrador Retriever	137	−0.363	0.309	−1.173	0.243	0.347
Mixed Breed	95	0.611	0.588	1.038	0.302	0.037
Other	84	2.031	0.519	3.914	0.000186	0.344
(B) chr16:5181388						
BREED	N	Beta	SE	T statistic	*P* value	Freq_A_allele
German Shepherd Dog	25	0.097	0.425	0.228	0.821	0.060
Golden Retriever	36	−0.219	0.130	−1.684	0.101	0.444
Labrador Retriever	137	−0.434	0.133	−3.277	0.0013	0.187
Mixed Breed	95	−0.325	0.166	−1.951	0.054	0.132
Newfoundland	15	−0.580	0.283	−2.052	0.061	0.367
Other	84	−0.268	0.116	−2.299	0.024	0.375

Only the major breeds in which all the alleles for each genotype were segregating are shown

## Discussion

This sample of purebred and mixed-breed dogs is a subset of dogs that were genotyped previously for complex and fixed trait mapping [29]. Among the dogs in the larger study, 921 had Norberg angle measurements and, of these, we measured pelvic dimensions on 392 dogs. Principal component 1, when analyzed with sex in the model as a covariate, was associated with a locus that marks *IGF-1*. Insulin-like growth factor-1 is a major local growth factor that mediates chondrocyte proliferation and hypertrophy [35] as well as osteoblast and osteoclast behavior. Two copies of the derived allele of *IGF-1* are associated with small stature in dogs [15, 36]. That male dogs possess larger pelves has been reported based on 20 metrics in Labrador Retrievers [37]. These authors then developed a predictive model based on these measurements that could be applied to discriminate a male from a female canine pelvis in forensic investigations. Most of the pelvis is formed and grows through a process of intramembranous ossification except the acetabulum, which also grows by endochondral ossification. Thus IGF-1 levels influence the size of the pelvic bones through its effects on ossification [38]. The strength of the association with PC1 was not markedly affected by exclusion of the related Greyhound/Labrador Retriever cross breeds because this association was driven by the dog breeds with lower representation (Table 6).

Principal Component 3, representing a trade-off between ilial length and ischial length is consistent with the shorter, broader breeds having wider bodies relative to their body height and length. Breeds with this morphotype, like the Bulldog, Pug, Basset Hound, American Bulldog, French Bulldog, and Pit Bull Terrier are ranked among the worst 30 breeds in frequency of HD in the Orthopedic Foundation for Animals (OFA) HD registry (http\offa.org) and are at higher risk than mixed breed controls [15]. In contrast, the breeds with the lowest frequency of HD in this registry are what Chase and Lark [14] describe as the gracile breeds: Irish Wolfhound, Belgian Tervuren, Irish Setter, Greyhound, Whippet, Italian Greyhound, Saluki, Collie, and Borzoi; breeds with longer ilia relative to the length of their ischiae. Additional file 4: Figure S2 indicates that the major effect of body weight was consumed by the weightings on PC1 and that PC3 was less affected by body weight because the breeds with the most dogs did not separate according to breed (which is related to body size).

The closest candidate gene to the PC3 locus on CFA16 (*P* = 1.9×10^−7^) is *TPK1*, a thiamine-dependent enzyme and part of the protein kinase A (*PKA*) complex. *TPK1* is a cellular enzyme, abundantly expressed in maternal, placental and fetal tissues [39], which catalyzes the conversion of thiamine, a form of vitamin B1, to thiamine pyrophosphate (TPP). TPP is an active cofactor for enzymes involved in glycolysis and energy production, including transketolase, pyruvate dehydrogenase, and alpha-ketoglutarate dehydrogenase [40]. Polymorphisms in this highly conserved enzyme *TPK1,* were associated with birth weight in neonates and the maternal genotype at the same locus also affected birth weight [40]. No studies are available that compare birth weight to subsequent occurrence of HD. Caloric restriction is known to reduce the incidence and severity of HD [41] and improved glucose handling due to restricted caloric intake improves longevity in dogs [42]. The mechanism of how growth rate influences these processes is unknown. Thiamine is essential for normal development of the nervous system and there is limited research linking muscle and nerve dysfunction in HD [43, 44].

Principal component 2, which was mostly weighted by the internal pelvic angle, had suggestive evidence of association. Dogs with greater acetabular coverage of the femoral head have better hip conformation than those with less coverage and are less prone to secondary OA, the hallmark of prior HD. Because the acetabulum is an integral component of the hemipelvis, we hypothesized that dogs with a more ventroverted hemipelvis would have higher internal pelvic angles and that this would be a feature of breeds resistant to HD. Our hypothesis is supported by analyses of computed tomographs (CT) of babies with developmental dysplasia of the hip (DDH) [45]. Fujii et al.,[35], examined 82 hips of 52 patients and concluded that structural abnormalities exist throughout the pelvis in DDH, and the morphologic abnormalities of the acetabulum are not caused solely by local dysplasia around the hip, but are influenced by the morphologic features of the entire pelvis. They observed greater internal rotation of the innominate bone in patients with DDH than in the control subjects along with increased acetabular anteversion angle and acetabular inclination angle. Internal rotation of the innominate bone also was associated with decreased anterior and superior acetabular coverage [45]. Perhaps the two-dimensional nature of a radiograph as suggested by one of the reviewers of our paper, lacked the finesse to capture the finer detail of pelvic rotation and additional measurements made from CT would be more sensitive.

We failed to identify polymorphisms associated with pelvic morphology as described by Chase et al., [20]. This may be due to their inclusion of limb morphology, as well as pelvic dimensions, in their PCA or our data set may not have attained sufficient power for associations with the other PCs to reach the genome-wide *P* value threshold. Chase et al., [20] conducted their morphologic mapping in a single breed, the Portuguese Water Dog. We used a population sample that consisted of many breeds. These breeds and the cross breeds were predominantly medium to large breed dogs. Seventeen QTLs can explain 80–88% of the variation of body weight and height in individual purebred dogs [29]. The same group also demonstrated that 500–1000 cases and controls are needed to uncover trait or disease loci of moderate effect. Thus, because the pelvic morphology is affected by size and body weight, which themselves are affected by more than 17 loci, it is unlikely that this study contains enough dogs to discover all loci of small to medium effect, especially when background genetics change across all the breeds in this study. Finally, we applied a stringent Bonferroni correction to the experiment-wide *P* value, some associations were hidden when compared to application of a more liberal false discovery rate.

However, PCA of 13 measurements of the pelvis across these breeds of dog provided evidence that *IGF-1* is associated with the overall size of the pelvis and, as expected, sex is related to pelvic size. We identified an association of the ilial/ischial proportion with a thiamine-dependent, candidate gene. The Norberg angle, a measure of HD in the dog, was moderately correlated with three PCs. Thus, the genes underlying these PCs may also predispose dogs to HD.

## Conclusions

Based on the current study, pelvic shape alone was not strongly associated with caninehip dysplasia.

## Additional files

Additional file 1: Table S1. Excel file of all pelvic measurements of 530 dogs. (XLSX 94 kb)

Additional file 2: Table S2. Excel file of the subset of 392 pelvic measurements used in this study. (XLSX 57 kb)

Additional file 3: Figure S1. PCA plot color-coded by breed (A with all breeds included, B with the four main breeds only). The correlation coefficient (r) between PC1 (the PC that is driven by weight) and body weight was 0.79. (PDF 190 kb)

Additional file 4: Figure S2. Box and whisker plots showing the distribution of PC values for the two significant associations across the five main breeds. (A) PC1, (B) PC3. (PDF 23 kb)

Additional file 5: Table S3. Genome wide association study was performed on the first five principal components after removing 142 of the most related (pi-hat > 0.5) Labrador Retriever, Greyhound, and Greyhound/Labrador Retriever (mix) dogs from the analysis. Chr is the chromosome number; bp is base pairs. (DOCX 13 kb)

## Abbreviations

GWAS: Genome wide association study
HD: Hip dysplasia
IGF-1: Insulin-like growth factor 1
MAF: Minor allele frequency
NA: Norberg angle
PC: Principal component
PCA: Principal component analysis
PKA: Protein kinase A
QTL: Quantitative trait locus/loci
SNP: Single nucleotide polymorphism
TPK1: Thiamine pyrophosphokinase 1
