# Supplementary figures and images for: Genetic mapping of principal components of canine pelvic morphology
Source: Canine Genet Epidemiol. 2017 Mar 24;4:4. doi: 10.1186/s40575-017-0043-7 (PMC5364603; doi:10.1186/s40575-017-0043-7)

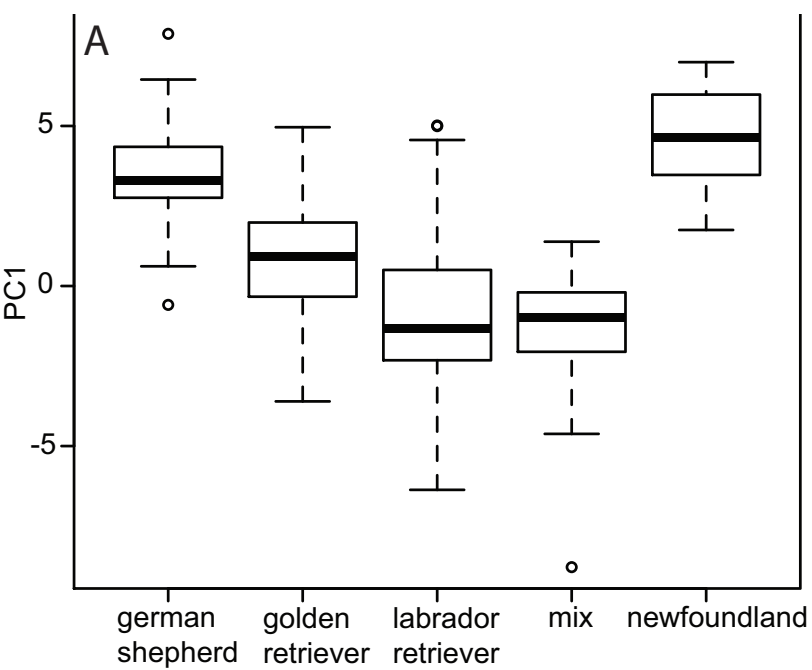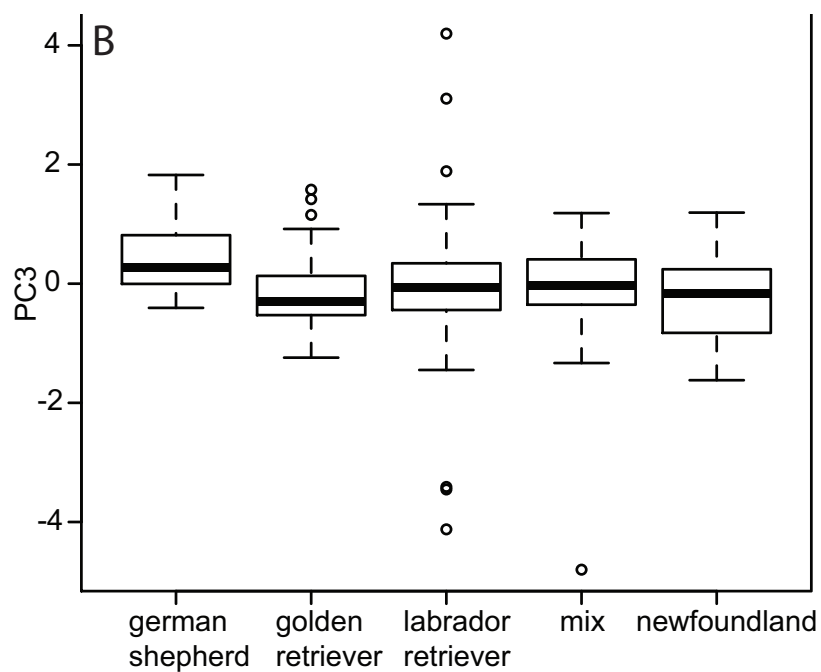

Supplement: Supplementary file 4 — Box and whisker plots showing the distribution of PC values for the two significant associations across the five main breeds. (A) PC1, (B) PC3. (PDF 23 kb) [file 40575_2017_43_MOESM4_ESM.pdf]
