# Supplementary material for: Genetic mapping of principal components of canine pelvic morphology
Source: Canine Genet Epidemiol. 2017 Mar 24;4:4. doi: 10.1186/s40575-017-0043-7 (PMC5364603; doi:10.1186/s40575-017-0043-7)
Supplement: Supplementary file 5 — Genome wide association study was performed on the first five principal components after removing 142 of the most related (pi-hat > 0.5) Labrador Retriever, Greyhound, and Greyhound/Labrador Retriever (mix) dogs from the analysis. Chr is the chromosome number; bp is base pairs. (DOCX 13 kb) [file 40575_2017_43_MOESM5_ESM.docx]

| Phenotype | Related dogs **included**  Chr:bp | Related dogs **included**  P-value | Related dogs **excluded**  Chr:bp | Related dogs **excluded**  P-value |
| --- | --- | --- | --- | --- |
| PC1 | 15:41229597 | 1.93x10^-8^ | 15:41229597 | 1.08x10^-8^ |
| PC2 | 26:15666332 | 5.75x10^-7^ | 26:15666332 | 1.94x10^-6^ |
| PC3 | 16:5181388 | 1.91x10^-7^ | 3:33109793 | 6.45x10^-6^ |
| PC4 | 5:36523896 | 2.47x10^-6^ | 21:19984047 | 2.41x10^-6^ |
| PC5 | 1:91463706 | 6.67x10^-7^ | 5:51013074 | 4.75x10^-7^ |
