# Supplementary material for: Genetic mapping of principal components of canine pelvic morphology
Source: Canine Genet Epidemiol. 2017 Mar 24;4:4. doi: 10.1186/s40575-017-0043-7 (PMC5364603; doi:10.1186/s40575-017-0043-7)
Supplement: Supplementary file 3 — PCA plot color-coded by breed (A with all breeds included, B with the four main breeds only). The correlation coefficient (r) between PC1 (the PC that is driven by weight) and body weight was 0.79. (PDF 190 kb) [file 40575_2017_43_MOESM3_ESM.pdf]

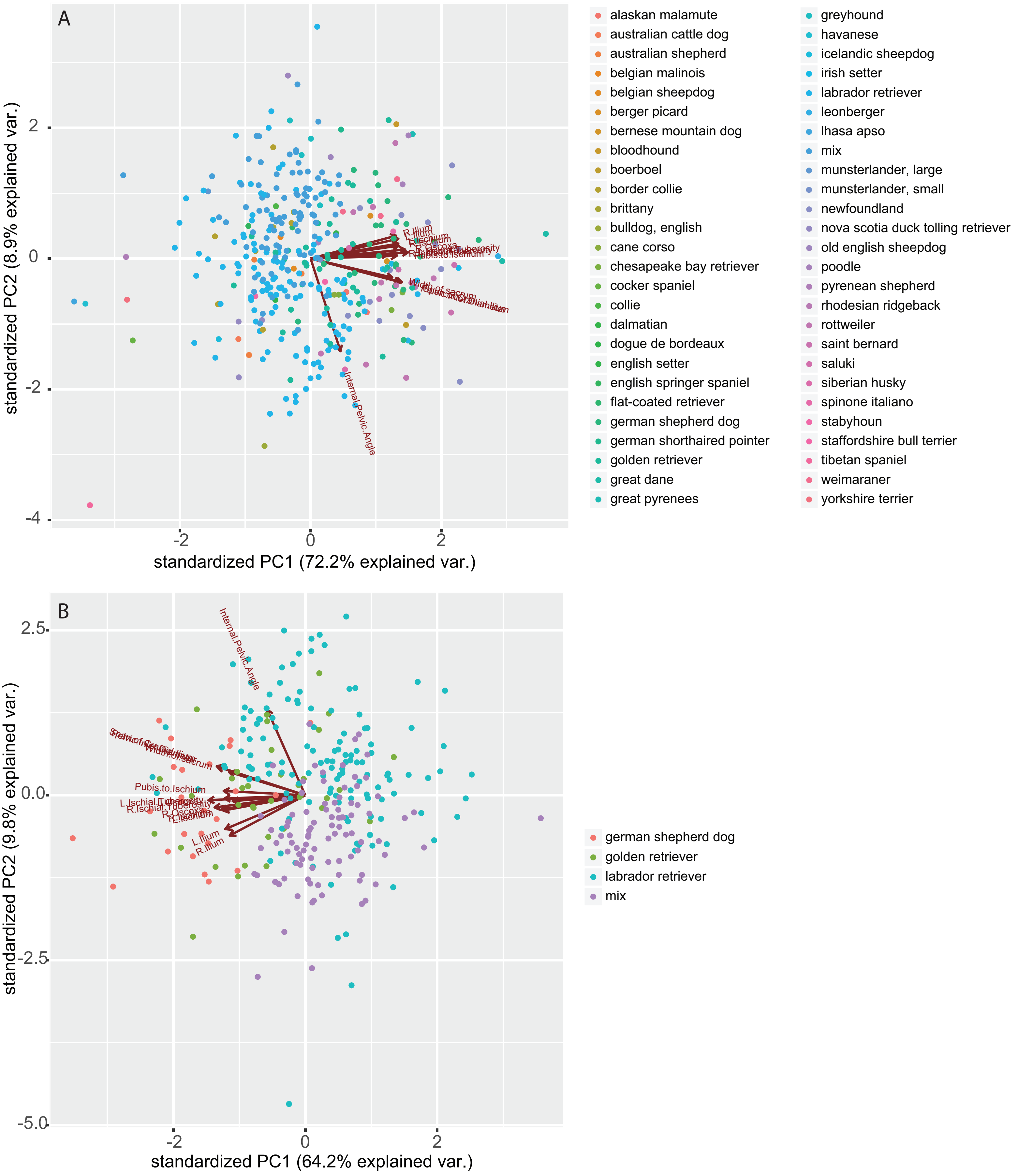

A
standardized PC2 (8.9% explained var.)
standardized PC1 (72.2% explained var.)
2
0
-2
-4
-2
0
2
R.Ilium
Width.of.sacrum
Internal.Pelvic.Angle
alaskan malamute
australian cattle dog
australian shepherd
belgian malinois
belgian sheepdog
berger picard
bernese mountain dog
bloodhound
boerboel
border collie
brittany
bulldog, english
cane corso
chesapeake bay retriever
cocker spaniel
collie
dalmatian
dogue de bordeaux
english setter
english springer spaniel
flat-coated retriever
german shepherd dog
german shorthaired pointer
golden retriever
great dane
great pyrenees
greyhound
havanese
icelandic sheepdog
irish setter
labrador retriever
leonberger
lhasa apso
mix
munsterlander, large
munsterlander, small
newfoundland
nova scotia duck tolling retriever
old english sheepdog
poodle
pyrenean shepherd
rhodesian ridgeback
rottweiler
saint bernard
saluki
siberian husky
spinone italiano
stabyhoun
staffordshire bull terrier
tibetan spaniel
weimaraner
yorkshire terrier
B
standardized PC2 (9.8% explained var.)
standardized PC1 (64.2% explained var.)
2.5
0.0
-2.5
-5.0
-2
0
2
Internal.Pelvic.Angle
Pubis.to.Ischium
L.Ischial.Tuberosity
R.Ischial.Tuberosity
R.Oscoxa
R.Ischium
L.Ilium
german shepherd dog
golden retriever
labrador retriever
mix
